# Protocol of a pilot-scale, single-arm, observational study to assess the utility and acceptability of a wearable hydration monitor in haemodialysis patients

**DOI:** 10.1186/s40814-022-00976-7

**Published:** 2022-01-24

**Authors:** Vicki Sandys, Colin Edwards, Paul McAleese, Emer O’Hare, Conall O’Seaghdha

**Affiliations:** 1 Royal College of Surgeons in Ireland, 123 St Stephen’s Green, Dublin 2, Ireland; 2 patientMpower Ltd., 21 Denzille Lane, Dublin, D02 EY19 Ireland; 3 Design to Value Ltd., Innishannon, Co., Cork, Ireland

## Abstract

**Background:**

Fluid overload has a high prevalence in haemodialysis patients and is an important risk factor for excess mortality and hospitalisations. Despite the risks associated with chronic fluid overload, it is clinically difficult to assess and maintain fluid status adequately. Current methods of fluid status assessment are either imprecise or time intensive. In particular, to date, no method exists to accurately assess fluid status during the interdialytic interval.

**Objectives:**

This pilot study aimed to evaluate whether a prototype wearable hydration monitor can accurately and reproducibly detect fluid overload in the haemodialysis population when compared to haemodialysis and bioimpedance data.

**Methods:**

A prospective, open-label, single-arm observational trial of 20 patients commenced in January 2021 in a single haemodialysis centre in Ireland, with a wearable hydration monitor, the Sixty device. The Sixty device uses diffuse reflectance spectroscopy to measure fluid levels at the level of the subdermis and uses machine learning to develop an algorithm that can determine fluid status. The Sixty device was worn at every dialysis session and nocturnally over a three-week observational period. Haemodialysis parameters including interdialytic weight gain, ultrafiltration volume, blood pressure, and relative blood volume were collected from each session, and bioimpedance measurements using the Fresenius body composition monitor were performed on 4 occasions as a comparator. The primary objective of this trial was to determine the accuracy and reproducibility of the Sixty device compared to bioimpedance measurements.

**Conclusion:**

If the accuracy of the wearable hydration monitor is validated, further studies will be conducted to integrate the device output into a multi-parameter machine learning algorithm that can provide patients with actionable insights to manage fluid overload in the interdialytic period.

**Trial registration:**

www.clinicaltrials.govNCT04623281. Registered November 10th, 2020.

## Background and rationale

Haemodialysis (HD) patients experience an excess of cardiovascular morbidity and mortality [1]. Volume overload is one modifiable risk factor for cardiovascular disease and hypertension in this population [2]. The non-physiological fluid shifts associated with intermittent, thrice-weekly dialysis exposes patients to cardiac stress. Chronic fluid overload can precipitate systemic [3] and pulmonary hypertension [4] and left ventricular remodeling [5], and contributes to hospitalisation [6] and mortality rates [7]. Conversely, rapid ultrafiltration rates required to correct excess volume can cause haemodynamic instability, leading to a range of complications including myocardial stunning, arrhythmias, and intradialytic hypotension [8].

Maintaining fluid is an integral aspect of haemodialysis care, but is difficult to perform adequately, as reflected in the high prevalence of fluid overload [9]. Traditional methods of assessing fluid, such as the clinical exam and blood pressure, are imprecise correlates of fluid status [10, 11]. Newer technologies, such as lung ultrasound, can detect subtle changes of volume overload [10], but are time and resource intensive [12, 13].

Bioimpedance is a validated method of estimating volume overload by directly assessing the extracellular water (ECW), intracellular water (ICW), and total body water (TBW) compartments, and compares well against gold standard methods [14], but is limited by expense, time, and availability [13]. More recent devices have incorporated multifrequency thoracic bioimpedance into wearable technology [15, 16]. The majority of these techniques to date are designed to facilitate physician-directed volume reduction. Few, if any, allow patients to track and adjust their volume status during the interdialytic period [17].

To date, no specific outpatient wearable hydration monitor for haemodialysis patients is available and validated. The purpose of this trial is to compare the reproducibility and accuracy of a wearable hydration monitor, the Sixty device, against standard haemodialysis parameters and bioimpedance measurements. If this hydration monitor is validated, it will contribute to the development of a multivariate algorithm to assist haemodialysis patients in monitoring and adjusting their fluid intake. To our knowledge, this approach has not yet been explored in haemodialysis patients.

## Objectives

This trial was conducted to evaluate if a wearable hydration monitor (the Sixty device) has validity and reproducibility against traditional and validated methods of volume assessment in haemodialysis patients. If the device is validated, further studies will be conducted to integrate the Sixty device data into a multi-parameter machine learning algorithm that can provide patients with actionable insights to manage fluid overload in the interdialytic period.

This was a pilot-scale study to assess if this approach has merit and could be tested and developed further. As no specific wearable hydration monitor for haemodialysis patients is available and validated, there was sufficient justification in testing the effectiveness and acceptability of the Sixty device in a controlled observational setting.

The primary objective was to evaluate the accuracy of the Sixty device in assessing volume in haemodialysis patients compared to bioimpedance measurements using the Fresenius body composition monitor (BCM).

### Primary outcome measure

1. Comparison of fluid status as measured by the Sixty device compared to bioimpedance measurements performed pre-dialysis by a body composition monitor (BCM; Fresenius).

### Secondary outcome measures

1. Comparison of changes in fluid status as determined by the Sixty device versus ultrafiltration millilitres/per unit time during a haemodialysis session
2. Comparison of changes in fluid status as determined by the Sixty device versus change in weight pre and post dialysis (kg)
3. Comparison of changes in fluid status as determined by the Sixty device versus change in blood volume monitoring (relative blood volume %)
4. Comparison of changes in fluid status as determined by the Sixty device versus blood pressure (mmHg)
5. The patient's opinion of the acceptability of the Sixty device as assessed by their response to a questionnaire.

The outcome measures were chosen as proxies of fluid status used in clinical practice.

## Trial design

**Fig. 1:**
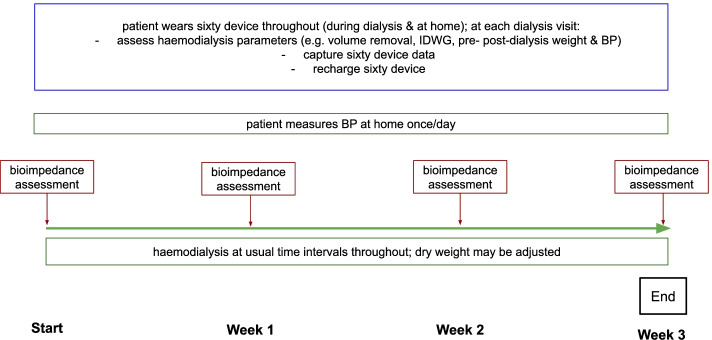
Diagram of trial design

This was a prospective, open-label, single-arm observational study with wearable hydration, the Sixty device, occurring over 3 weeks in a single centre at Beaumont Hospital, a tertiary care centre for nephrology in Dublin, Ireland (Fig. 1).

An estimated 20 patients were recruited. All patients were allocated to the same observation sequence. Patients followed their usual haemodialysis regimen throughout the study. Patients’ dry weights were optimised using bioimpedance measurements during the study.

Patients were asked to wear a wearable hydration monitor on their forearm during haemodialysis sessions and nocturnally. Clinical assessments (e.g. IDWG, BP, pulse rate, ultrafiltration volume, and symptoms) were assessed at each dialysis visit. Bioimpedance measurements using the body composition monitor (Fresenius BCM) were performed at the start of the observation period, and once a week before the mid-week dialysis session.

## Methods: Participants, interventions, and outcomes

### Selection of trial population

Approximately 300 patients attend for haemodialysis in an ambulatory care setting under the governance of Beaumont Hospital. Eligible individuals were invited to participate. Patients with a range of baseline fluid status, age, and gender were considered for enrolment. Written informed consent was obtained for each patient prior to starting the study. Twenty [18] people were enrolled. The sample size was chosen arbitrarily.

### Inclusion criteria

Participants were able to enrol in the study if they were attending the haemodialysis unit for haemodialysis at least two to three times a week, and if all of the following applied at initial consent: aged at least 18 years; demonstrates understanding of correct use of the Sixty device; willing to give written informed consent.

### Exclusion criteria

Participants were excluded from the study if there were conditions precluding accurate use of bioimpedance such as the presence of an implantable cardioverter defibrillator, pacemaker, hearing aids, or pregnancy. Patients were furthermore excluded if there was significant confusion or any concomitant medical condition, which would limit the ability of the patient to record symptoms or other parameters.

### Recruitment

Patients’ consent was sought during the normal haemodialysis treatment sessions. Patients were provided with a plain-language patient information leaflet related to the Sixty device, as well as a consent form explaining the study rationale, processes, data storage procedure, and the contact details for researchers. Consent was obtained from patients by the co-investigators of the trial, or a suitable health research personnel nominee. Recruitment was continued until the planned sample size was achieved.

### Removal of individual patients and discontinuation of the trial

Patients were free to withdraw from the study at any time without any impact on their ongoing medical care.

The investigator could withdraw a patient from the study at any time if they believed that further participation in the study was not in the best interests of the patients. The study could be terminated early if recruitment was significantly behind schedule or if for any other reason, it was unlikely that the study would be completed.

## Description of intervention and comparator

**Fig. 2:**
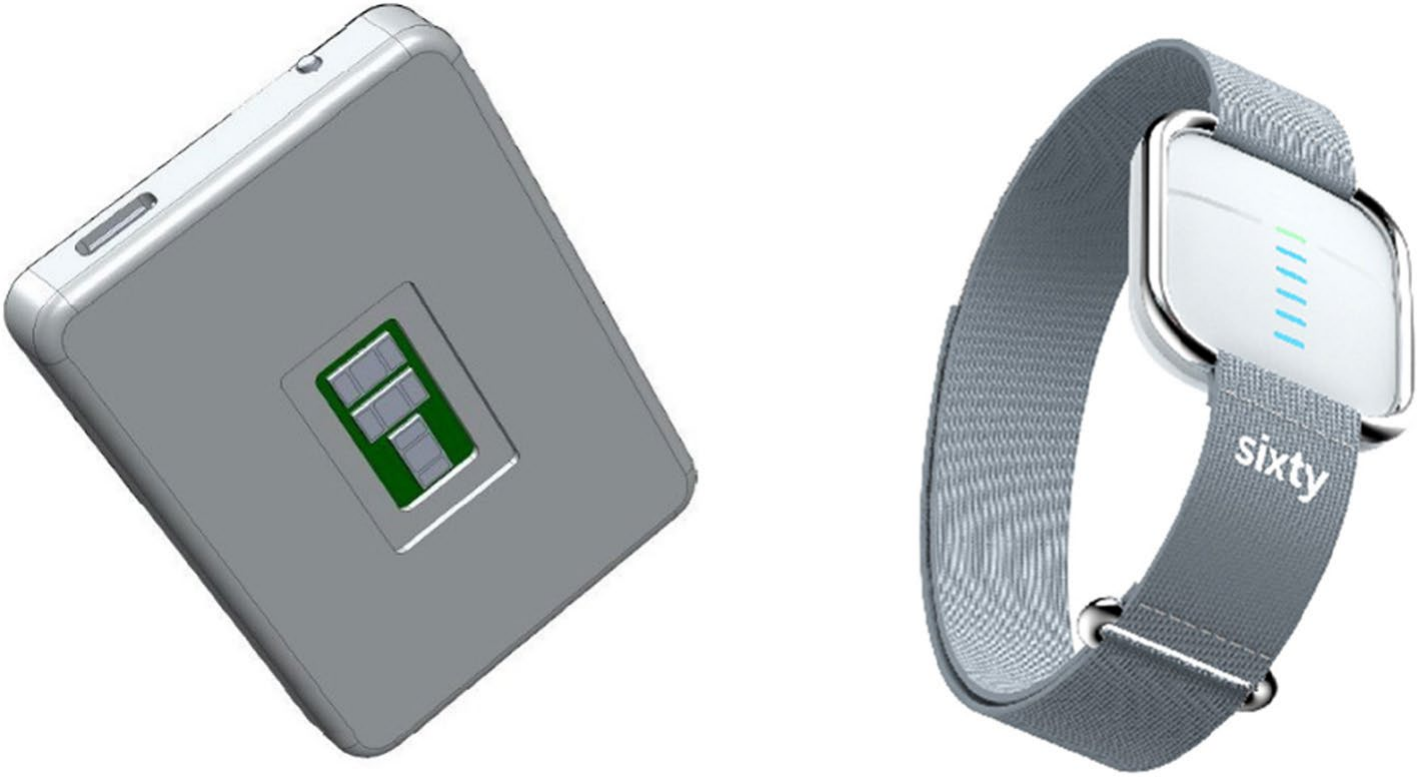
Photographs of prototype and expected final design of the Sixty device

The Sixty device is a wearable hydration monitor that is currently in development by Design to Value Ltd. Photographs of the current prototype and expected final designs are shown in Fig. 2.

The prototype device (left-hand image) measures 66mm × 48mm × 10mm. The on/off button and charging port are visible on the top edge. The surface facing the camera was in contact with the dorsal surface of the user’s wrist. The expected final design is shown in the right-hand image. This is expected to be approximately the size of a commercially available smartwatch.

The Sixty device combines photonics sensors and the principle of diffuse reflectance spectroscopy to determine fluid status. The sensors include red, green, and infrared wavelengths in the range of 530 to 950nm. The amount of light reflected back can be correlated with the concentration of molecules within the tissue (e.g. water). The algorithm has been trained using a calibrated dataset of known fluid states that allows the device to interpret the data and provide feedback on fluid status in kilogrammes and/or litres. As the algorithm has previously been trained on healthy volunteers, a new algorithm was developed for the prediction of fluid states in haemodialysis patients.

The prototype device used in this study does not reflect the final aesthetic, but provides the necessary functionality to establish proof of principle in fluid overloaded patients. The prototype device only collected data and did not provide feedback to the user on their hydration status. Data was collected locally on a memory card (i.e. SD card) which was removed periodically to recover the data.

The device and battery are designed to run for an extended period and can be charged via a USB cable. Charging the device and collection of data were coordinated at the haemodialysis study centre to minimise data gaps.

### Body composition monitor

The body composition monitor is a whole-body bioimpedance device that uses a multi-frequency current to measure body compartments, such as intracellular water (ICW), extracellular water (ECW), and total body water (TBW) through electrodes placed on a parallel wrist and ankle [19]. In addition, the BCM device calculates normally hydrated lean and adipose tissue in order to account for the effect of body composition on ECW. As the extracellular water component of total body water is thought to contribute predominantly to the signs and symptoms of fluid overload, the overhydration index (OH) is calculated as the difference between the measured ECW and the normal ECW as determined by body composition [14].

BCM is a reliable, validated method of fluid assessment that has been validated against gold standard dilution methods of volume assessment across multicentre studies in both healthy patients and haemodialysis patients, such as sodium bromide (ECW), deuterium and tritium (TBW) and total body potassium (ICW) [14]. Good agreement has been illustrated with reference methods including total ultrafiltration volume and dual-energy X-ray absorptiometry (DEXA) [19]. As such, BCM is the closest method to the gold standard we currently have to evaluate fluid status in nephrology patients.

## Data collection, management and analysis

The trial procedures at each visit are summarized in the flow chart (Table 1).

**Table 1:** Flow chart of assessments during the observation period

	Study observation period (3 weeks)				
	Baseline clinic Visit	At each dialysis session	Week 1	Week 2	Week 3 (end)
Informed consent	X				
Demographic data (include dialysis and medicine history)	X				
Vital signs, body weight, routine hemodialysis measurements in-clinic	X	X	X	X	X
Assess fluid status with bioimpedance	X		X	X	X
Instruction/patient training on the use of the Sixty device	X				
Wear the Sixty device daily	X	X	X	X	X
Download data and recharge the Sixty device		X	X	X	X
Record symptoms related to fluid status	X	X	X	X	X
Record adverse events	X	X	X	X	X
Patient records blood pressure at home once/day	Once/day throughout the study				
Stop using the Sixty device					X
Acceptability of the Sixty device questionnaire					X
End of study					X

### Baseline clinic visit

Following consent, demographic data, medical history and concomitant medications were obtained directly from the patient or from their electronic record (eMED).

At the baseline visit and at each haemodialysis visit during the study the following usual care parameters were assessed and recorded directly from the patient and nursing staff and from their dialysis electronic record (eMED). Haemodialysis session data was downloaded directly from the dialysis online documentation system; the Fresenius Therapy Monitor (TMON). Symptoms, adverse events, and requirement for additional unscheduled dialysis were captured on case report forms, filed in the trial documentation on-site.

Haemodialysis session variables:

- Total fluid removal (each haemodialysis session)
- IDWG
- Proportion of haemodialysis sessions in which IDWG is ≤ 4%
- Pre-dialysis and post-dialysis weight
- Pre-dialysis, intradialytic, and post-dialysis BP
- Pulse rate
- Relative Blood Volume (RBV %)
- Requirement for additional nursing intervention (e.g. stopping dialysis, administering fluid bolus)
- Adverse events related to the patient’s underlying condition (e.g. access-related complications, hypertension-related symptoms, congestive heart failure, pulmonary edema)
- Requirement for additional unscheduled dialysis, or need for hospitalisation
- Number and types of BP medication(s)

The research team allocated a Sixty device to the patient and instructed them on the correct use of the device. The patient wore the Sixty device and switched it on before the start of haemodialysis at this clinic visit. The patient wore the Sixty device throughout the haemodialysis session at this and all subsequent haemodialysis sessions during the study observation period as well as nocturnally.

### Observation period (weeks 1–3)

At each clinic visit, before the patient started haemodialysis, the research team removed the SD card from the patient’s Sixty device and downloaded the data collected in the previous 2–3 nights. The SD card was re-inserted into the Sixty device and the patient wore the Sixty device during the haemodialysis procedure. If necessary, the Sixty device was recharged during haemodialysis and continued to collect data while recharging.

The measurements related to haemodialysis and described previously (usual care haemodialysis parameters and symptoms) were recorded at each haemodialysis visit during the study observation period.

Fluid status was assessed by bioimpedance immediately before haemodialysis on four occasions, once on the most fluid overloaded day following the long interdialytic interval. Thereafter, bioimpedance measurements were performed once a week prior to the mid-week dialysis session.

Symptoms of hyper and hypovolaemia occurring within the past week were recorded once a week.

Symptoms of hypovolaemia:

- Thirst directly after haemodialysis
- Symptomatic hypotension
- Nausea and vomiting
- Muscle cramps
- Limpness/tiredness between dialysis sessions
- Dizziness between dialysis sessions

Symptoms of hypervolaemia:

- Chronic coughing (new)
- Dyspnea on exertion
- Dyspnea at rest; 1 pillow
- Dyspnea at rest: 2 pillow
- Dyspnea at rest: 3 pillows
- Pretibial oedema
- Paroxysmal nocturnal dyspnea (PND)

The patients completed a questionnaire to give their opinion on the acceptability of the Sixty device. This was assessed and recorded following the end of the week 3 observation period.

Examples of questions in the acceptability questionnaire include:

- How do you currently manage your fluid intake in between dialysis sessions?
- On a scale of 1 to 5, how well do you feel in control of fluid status during this interdialytic period? (1 meaning “no control”... with 5 meaning “fully in control with good understanding what triggers fluid overload”)
- Would you wear a device similar in looks to a FitBit/Apple watch, if it could continuously monitor your fluid status and prompt you when and how much to drink to stay hydrated yet limit fluid overload?
- What is the minimum acceptable functionality that this device would need to have in order for you to wear it (check box list of features such as):
  - Tells me my hydration level and prompts me on how to manage fluid intake
  - Tells the time
  - Measures heart rate
  - Measures steps/activity
  - Measures sleep quality

The study procedures were concluded at this visit.

## Efficacy endpoints

### Primary endpoint

The primary objective was to evaluate the accuracy and reproducibility of the Sixty device in assessing volume in haemodialysis patients compared to bioimpedance using the Fresenius body composition monitor (BCM).

The primary endpoint variables included:

1. Comparison of fluid status as measured by Sixty device compared to bioimpedance measurements using BCM.
  Volume overload as defined by bioimpedance
  Relative overhydration: Overhydration (OH)/Extracellular water (ECW) > 7%
  Overhydration index: ≥ 1.1L

- Normohydration is defined as OH/ECW − 7 to + 7% (corresponding to OH index of − 1.1 L to + 1 L)
- Dehydration as defined by bioimpedance
  - Relative OH < − 7%
  - OH index ≤ − 1.1L

Secondary outcome measures:

1. Comparison of changes in fluid status as determined by the Sixty device versus ultrafiltration millilitres/per unit time during a haemodialysis session.
2. Comparison of changes in fluid status as determined by the Sixty device versus change in weight pre and post dialysis (kg)
3. Comparison of changes in fluid status as determined by the Sixty device versus blood pressure (mmHg)
4. Comparison of changes in fluid status as determined by the Sixty device versus change in blood volume monitoring (Relative blood volume %)
5. The patient’s opinion of the acceptability of the Sixty device as assessed by their response to a questionnaire.

An accuracy of > 70% in predicting fluid status categories as measured by BCM and/or evidence of good correlation with BCM readings as indicated by a correlation coefficient > 0.70 will be considered sufficient justification for further trials. In the absence of the above, evidence of high correlation with ultrafiltration volume (> 0.70) will be considered reasonable grounds for further assessment of the device.

## Data management

All endpoint data was stored on a central database for analysis. The data as reported by the patients was queried before descriptive statistical analysis tables were prepared. Patients are identified by a unique identification number on the study database (i.e. data is pseudonymised). Each patient’s data is linked to their unique identification number.

The original electronic data and relevant medical records were the source documents. The research team ensured that all data entered into the central database was a true record of events. Source data verification was not performed. Medical data relating to patient care has been stored in the medical records according to the usual procedures of the treatment site(s).

The data processor for this study is patientMpower Ltd., a digital health company. The endpoint data recorded on the Sixty device, haemodialysis parameters (including bioimpedance data), and symptoms data is stored on a central database managed by patientMpower Ltd. Pseudonmyised data was transferred to patientMpower via a secure Amazon Web Service server. The pseudonymised data was shared with the Beaumont Hospital research team, Design to Value Ltd. (developer and owner of the Sixty device), and patientMpower Ltd.

The trial was completed when 20 patients completed the 3-week observation period.

All data was analysed on an intention to treat basis without regard to protocol violations.

## Data analysis

Patient demographic data will be reported as means and standard deviations or frequencies and proportions. “A proprietary algorithm is used by Design to Value Ltd. (developer of the Sixty device) to develop personalised determination of fluid status based upon the raw data from the Sixty device. The algorithm is capable of outputting an outcome concordant with fluid status in kilogrammes and/or litres. Haemodialysis sessions per patient will be divided into training (approx. 2/3) and test (1/3) sets to develop and test the Sixty machine learning algorithm. Reproducibility of the algorithm will be evaluated by evaluating the algorithm’s performance on the test dataset. Training of the algorithm will be performed by Design to Value Ltd., developers of the Sixty device. Evaluation of the algorithm on the test set will be performed by the clinical research team. The primary and secondary objectives will be analysed by the clinical research team.

The primary objective will be assessed by examining the agreement or correlation between the Sixty hydration score within the first 20 min and last 20 min and BCM measures of fluid status including the overhydration index (OH) pre and post dialysis. This will assess the accuracy of the device algorithm.

The change in Sixty hydration readings from start to end in dialysis (kg) will be calculated and compared with the change in ultrafiltration volume (l). The correlation and root mean squared error (RMSE) for the fluid change (i.e. the Sixty start–end in kilogrammes against the total ultrafiltration volume in litres) will be calculated on training and test data separately. A *t*-test will be calculated to determine if the fluid changes are statistically different.

The interdialytic change in the Sixty hydration value in kilogrammes for the start of the current dialysis session minus the end of the previous dialysis session will be calculated and compared to the interdialytic weight gain. The correlation and RMSE for the interdialytic change (as described above for Sixty during dialysis and dialysis weight) will be calculated and a t-test will be applied to assess for statistical significance.

The Sixty hydration value in kilogrammes for the start of dialysis, end of dialysis, and the change (start–end value) will be compared to the pre-dialysis weight, post-dialysis weight and pre- minus post-dialysis weight respectively using correlation and RMSE, and a *t*-test for statistical difference.

Nocturnal Sixty data will be evaluated by (1) assessing if there is a statistical difference between the means and standard deviations of nights one to three; (2) assessing if there is a statistical difference between the Sixty value at the end of a dialysis session and the Sixty value start of the nearest night and, similarly, for Sixty values at the start of a dialysis session versus Sixty values in the morning, following nocturnal wear; and (3) assessing if there is a statistical difference between the change in Sixty hydration values from start to end of dialysis versus change in Sixty hydration values overnight.

A secondary analysis will determine the association between the Sixty hydration score and other correlates of volume including BP, RPV, and symptoms.

The acceptability and utility of the Sixty device will be assessed by analysis of the responses to a patient questionnaire.

Patient-reported symptoms and adverse events will be tabulated and displayed. Any additional healthcare resource utilisation (e.g. additional unplanned haemodialysis sessions, hospitalisation) will be tabulated and displayed. No imputations of missing data will be made.

## Safety

No safety issues were anticipated from the use of the Sixty device or bioimpedance assessments. Issues regarding abnormal fluid assessments were addressed by the nephrology department at Beaumont Hospital.

Any adverse events observed with medical treatments were reported to the manufacturers or suppliers of those treatments.

## Discussion

Fluid overload, fluid depletion, and fluid status variability are independent risk factors for cardiovascular events and mortality in hemodialysis patients [2, 20]. Maintaining volume within a controlled range is integral to haemodialysis care. In a retrospective study of 41,114 prevalent HD patients, high-amplitude fluctuations in fluid status over the preceding 6 months were associated with the highest risk for all-cause and cardiovascular mortality, compared to patients who were normovolaemic, or consistently fluid depleted or overloaded [20]. Chronic fluid overload not only affects patient morbidity and mortality, but has an impact on hospital resources. Fluid overload precipitates hospitalisation and readmission rates. In a study of 176,790 HD patients, 14% of patients required admission for 1 or more episodes of fluid overload, heart failure, or pulmonary oedema over 2.5 years, at a total cost of $266 million [6].

No universal standard for evaluating fluid status in dialysis patients exists. Clinical assessments of fluid, such as signs, symptoms, and BP measurements, lack the precision to detect subtle changes in volume status [9, 18]. The LUST trial demonstrated the ability of lung ultrasound to pick up lung congestion not evident on clinical examination [10]. Similarly, using bioimpedance measurements to target euvolaemia can improve overall fluid status and reduce BP [21–23] compared to clinical exam [21]. Devices such as the BIOZ system [15] and the COVA monitoring system [15, 16] use longitudinal intradialytic thoracic impedance measurements that have demonstrated a good correlation with ultrafiltration volumes. However, these techniques provide a peri-dialytic assessment of fluid only and rely on physicians for interpretation of results and fluid status adjustments.

To our knowledge, no device exists yet that can monitor patients’ fluid status interdialytically, during the outpatient period. If this wearable hydration monitor is validated, further studies will be conducted to integrate the Sixty device output into a multi-parameter machine learning algorithm that can provide patients with actionable insights into their fluid status. This algorithm will be tested prospectively in an observational study examining the use of digital health in fluid maintenance in dialysis patients.

## Data Availability

The datasets generated and/or analysed during the current study are not publicly available due to a 24-month embargo on certain data from the date of publication to allow for the commercialisation of research findings. Derived data supporting the findings of this study are available upon reasonable request from the corresponding author(s).

## Abbreviations

BP: Blood pressure
BCM: Body composition monitor
ESRD: End-stage renal disease
ECW: Extracellular water
ICW: Intracellular water
HD: Haemodialysis
HOPE: Haemodialysis Outcomes & Patient Empowerment
IDWG: Interdialytic weight gain
OH: Overhydration index
PND: Paroxysmal nocturnal dyspnea
RPV: Relative plasma volume
TBW: Total body water
TMON: Therapy monitor
UFR: Ultrafiltration rate
